# Restraint Stress-Induced Neutrophil Inflammation Contributes to Concurrent Gastrointestinal Injury in Mice

**DOI:** 10.3390/ijms25105261

**Published:** 2024-05-11

**Authors:** Rina Munalisa, Te-Sheng Lien, Ping-Yeh Tsai, Der-Shan Sun, Ching-Feng Cheng, Wen-Sheng Wu, Chi-Cheng Li, Chi-Tan Hu, Kuo-Wang Tsai, Yungling Leo Lee, Yu-Chi Chou, Hsin-Hou Chang

**Affiliations:** 1 Department of Molecular Biology and Human Genetics, Tzu-Chi University, Hualien 970, Taiwan; 108727110@gms.tcu.edu.tw (R.M.); alan211@mail.tcu.edu.tw (T.-S.L.); 108712109@gms.tcu.edu.tw (P.-Y.T.); dssun@mail.tcu.edu.tw (D.-S.S.); 2 Department of Pediatrics, Taipei Tzu Chi Hospital, Buddhist Tzu Chi Medical Foundation, New Taipei City 231, Taiwan; chengcf@mail.tcu.edu.tw; 3 Institute of Biomedical Sciences, Academia Sinica, Taipei 115, Taiwan; leolee@ibms.sinica.edu.tw; 4 Division of General Surgery, Department of Surgery, Hualien Tzu Chi Hospital, Buddhist Tzu Chi Medical Foundation, Hualien 970, Taiwan; 5 Department of Laboratory Medicine and Biotechnology, College of Medicine, Tzu Chi University, Hualien 970, Taiwan; 6 Department of Hematology and Oncology, Hualien Tzu Chi Hospital, Buddha Tzu Chi Medical Foundation, Hualien 970, Taiwan; 7 Center of Stem Cell & Precision Medicine, Hualien Tzu Chi Hospital, Buddha Tzu Chi Medical Foundation, Hualien 970, Taiwan; 8 Research Center for Hepatology and Department of Gastroenterology, Hualien Tzu Chi Hospital, Buddhist Tzu Chi Medical Foundation, Hualien 970, Taiwan; 9 Department of Research, Taipei Tzu Chi Hospital, Buddhist Tzu Chi Medical Foundation, New Taipei City 231, Taiwan; tch33225@tzuchi.com.tw; 10 College of Public Health, China Medical University, Taichung 404, Taiwan; 11 Biomedical Translation Research Center, Academia Sinica, Taipei 115, Taiwan; chou0315@sinica.edu.tw

**Keywords:** neutrophil extracellular trap, NETosis, restraint stress, gastrointestinal injury, activating transcription factor 3

## Abstract

Psychological stress increases risk of gastrointestinal tract diseases. However, the mechanism behind stress-induced gastrointestinal injury is not well understood. The objective of our study is to elucidate the putative mechanism of stress-induced gastrointestinal injury and develop an intervention strategy. To achieve this, we employed the restraint stress mouse model, a well-established method to study the pathophysiological changes associated with psychological stress in mice. By orally administering gut-nonabsorbable Evans blue dye and monitoring its plasma levels, we were able to track the progression of gastrointestinal injury in live mice. Additionally, flow cytometry was utilized to assess the viability, death, and inflammatory status of splenic leukocytes, providing insights into the stress-induced impact on the innate immune system associated with stress-induced gastrointestinal injury. Our findings reveal that neutrophils represent the primary innate immune leukocyte lineage responsible for stress-induced inflammation. Splenic neutrophils exhibited elevated expression levels of the pro-inflammatory cytokine IL-1, cellular reactive oxygen species, mitochondrial burden, and cell death following stress challenge compared to other innate immune cells such as macrophages, monocytes, and dendritic cells. Regulated cell death analysis indicated that NETosis is the predominant stress-induced cell death response among other analyzed regulated cell death pathways. NETosis culminates in the formation and release of neutrophil extracellular traps, which play a crucial role in modulating inflammation by binding to pathogens. Treatment with the NETosis inhibitor GSK484 rescued stress-induced neutrophil extracellular trap release and gastrointestinal injury, highlighting the involvement of neutrophil extracellular traps in stress-induced gastrointestinal inflammation. Our results suggest that neutrophil NETosis could serve as a promising drug target for managing psychological stress-induced gastrointestinal injuries.

## 1. Introduction

Living a high-stress lifestyle in modern times may result in gastrointestinal (GI) diseases [1,2]. Studies have linked chronic psychological stress and psychological disorders to an elevated risk of GI tract diseases [3,4,5,6]. The gut microbiota–brain axis plays a role in the connection between psychological stress and GI disorders. This axis involves multiple systems, such as the central nervous system, neuroendocrine system, immune system, enteric microbiota, and GI system [7,8]. However, changes in the gut microbiota usually take several days or weeks to occur [2,7,9,10]. Therefore, this axis may not fully explain how the acute GI diseases caused by mental stress that occur within hours develop in humans and experimental animals [11]. As a result, the exact mechanism by which acute psychological stress leads to GI injuries within hours remains poorly understood.

The restraint stress mouse model is a well-established technique used to investigate the effects of psychological stress on biochemical, physiological, and behavioral changes in mice [12,13,14]. As Evans blue is a dye that cannot be absorbed by the GI tract, orally administering it to mice will result in limited appearance in the plasma [15,16]. By examining the levels of plasma Evans blue over time, we can track stress-induced GI leakage in live animals and then examine disease progression at the molecular and cellular levels [15,16]. For instance, our use of this model revealed that acute restraint stress causes gut epithelial cell death and GI leakage, while platelets and activating transcription factor 3 (ATF3) play protective roles against these stress-induced defects [15,16]. As ATF3 is an anti-inflammatory transcription factor [17], and platelets are known to have anti-inflammatory functions [18,19], our results suggest that excessive inflammation is involved in the initiation of acute psychological stress-induced GI injury [15,16]. Nevertheless, the exact mechanism underlying acute psychological stress-induced inflammatory responses remains unclear.

The spleen is a major lymphoid organ located near the GI system that regulates both innate and adaptive immune functions [20,21]. Changes in spleen function have been linked to the development of inflammatory diseases [22], including those affecting the GI system [23]. Moreover, the spleen is involved in changes to the distribution of blood leukocytes in mice induced by restraint stress [24]. Nevertheless, the pathophysiological alterations in splenic leukocytes after acute psychological stress are not yet fully understood.

This report focuses on the analysis of innate immune leukocytes in the spleen, including neutrophils (Ly6G^+^), monocytes (CD11b^+^), macrophages (F4/80^+^), and dendritic cells (CD11c^+^). Our findings suggest that neutrophils are the primary lineage responsible for inflammation following restraint stress. After stress exposure, neutrophils exhibited higher levels of pro-inflammatory cytokine interleukin-1 (IL)-1, reactive oxygen species (ROS) from both cellular and mitochondrial sources, and cell death than the other leukocyte lineages. Our analysis of regulated cell death responses (RCDs) revealed that the formation of neutrophil extracellular traps (NETs) through NETosis is the primary cell death response of splenic neutrophils, as opposed to other forms of RCD such as apoptosis, pyroptosis, necroptosis, ferroptosis, and autophagy. NETs are extracellular DNA–protein complexes that bind to pathogens and modulate inflammation [25,26]. Treatment with the NETosis inhibitor GSK484 rescued stress-induced GI injury, suggesting that molecular pathways contributing to NET release could be potential drug targets for managing stress-induced GI injury.

## 2. Results

### 2.1. Restraint Stress Primarily Affects Neutrophils in the Spleen of Mice

**Figure 1:**
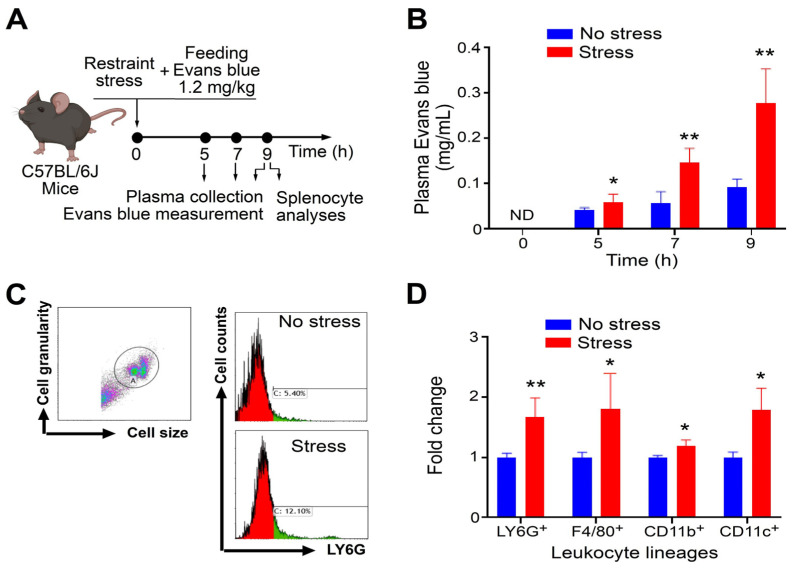
Restraint stress-induced gastrointestinal (GI) leakage is associated with the infiltration of innate immune leukocytes in the spleen of mice. (**A**) Experiment outline. (**B**) Plasma Evans blue levels, indicating the stress-induced GI leakage and injury levels. ND: not detected. (**C**) An example of the flow cytometry gating of Ly6G^+^ leukocytes after 9 h stress; the same method is applied to the analyses in (**D**). In (**C**), the letters A and C represent the gated total live splenic leukocytes and the LY6G^+^ cell population, respectively. (**D**) Relative cell-number levels of different leukocyte lineages (Ly6G^+^ neutrophil, F4/80^+^ macrophage, CD11b^+^ monocyte, CD11c^+^ dendritic cell) without (no stress groups) and with (stress groups) stress in the spleen of mice. The levels of respective leukocyte lineages in the no stress groups were normalized to one-fold. (**B**,**C**) * *p* > 0.05, ** *p* > 0.01, vs. no stress groups. *n* = 6 (3 experiments with a total of 6 mice per group). The mouse graph was created with BioRender.com, accessed on 1 January 2024.

In this investigation, we utilized a mouse model that underwent restraint stress and received the oral administration of Evans blue dye to evaluate the acute phase of gastrointestinal (GI) leakage, following the previously outlined methodology [15,16,27]. Using this model, we have demonstrated that acute restraint stress effectively disrupts epithelial cell tight junctions and leads to cell death [15,16]. The potential leakage of luminal substances from the gut into the circulation and abdominal lymphoid system, including the spleen, could theoretically result in inflammation and anomalous changes in the spleen. Hence, our aim was to examine the levels of innate immune leukocytes such as neutrophils (Ly6G^+^), macrophages (F4/80^+^), monocytes (CD11b^+^), and dendritic cells (CD11c^+^) in the spleens of mice. Our findings indicated that stress-induced GI leakage (Figure 1A, experimental outline; Figure 1B, plasma Evans blue levels) correlated with increased levels of innate immune leukocyte populations, including neutrophils, macrophages, monocytes, and dendritic cells (Figure 1C, gating; Figure 1D). These results suggest that stress-triggered pathophysiological changes involve modifications in the spleen, leading to elevated levels of innate immune leukocytes.

**Figure 2:**
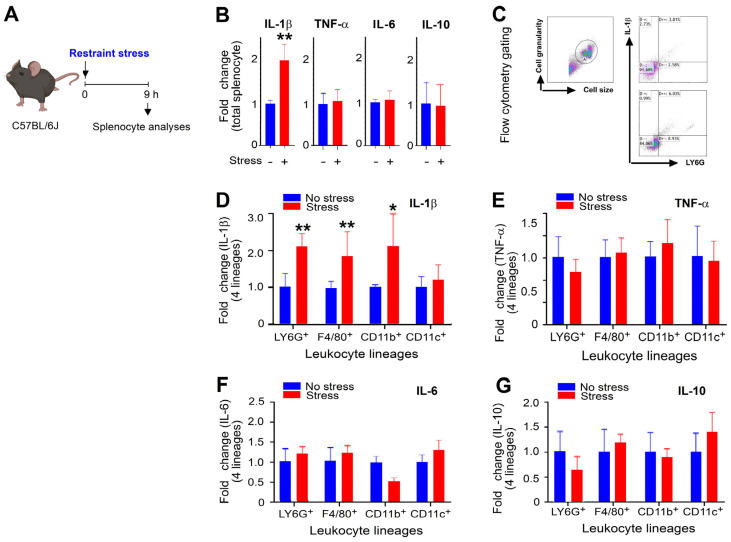
Restraint stress on the regulation of the cytokine expression of splenic innate immune leukocytes in mice. (**A**) Experiment outline. Blue: no stress groups; Red: stressed groups. (**B**) Relative levels of spleen splenocytes expressing pro-inflammatory cytokines IL-1β, TNF-α, IL-6, and anti-inflammatory cytokine IL-10, in mice with (stress groups) and without (no stress groups) 9 h stress. The levels of respective leukocyte lineages in the no stress groups were normalized to one-fold. (**C**) An example of flow cytometry gating for analyzing IL-1 expression in Ly6G^+^ leukocytes; the same method is applied to the analyses in (**C**–**F**). (**C**–**F**) Relative expression levels of IL-1β (**D**), TNF-α (**E**), IL-6 (**F**), IL-10 (**G**) in different leukocyte lineages (Ly6G^+^ neutrophil, F4/80^+^ macrophage, CD11b^+^ monocyte, CD11c^+^ dendritic cell) without (no stress groups) and with (stress groups) stress. The expression percentage of tested cytokines was normalized to a baseline value of 1 for the respective leukocyte lineage population in the no stress groups. (**B**,**D**–**G**) * *p* > 0.05, ** *p* > 0.01, vs. no stress groups; *n* = 6 (3 experiments with a total of 6 mice per group). The mouse graph was created with BioRender.com, accessed on 1 January 2024.

Pro-inflammatory cytokines interleukin (IL)-1β, tumor necrosis factor-α (TNF-α), and IL-6, as well as anti-inflammatory cytokine IL-10, are known to be up-regulated in various inflammatory diseases [28,29,30,31,32]. In this study, we analyzed the expression levels of these cytokines in total splenic leukocytes to investigate the inflammation status of the cells. Our results showed that only IL-1β was markedly up-regulated after stress (Figure 2A, experiment outline; Figure 2B, stress vs. no stress groups ** *p* < 0.01; IL-1β, vs. TNF-α, IL-6, IL-10). Further lineage analysis revealed that IL-1β expression was up-regulated in splenic Ly6G^+^ neutrophils, F4/80^+^ macrophages, and CD11b^+^ monocytes but not in CD11c^+^ dendritic cells (Figure 2C, flow cytometry gating; Figure 2D, relative IL-1 signals in respective leukocyte lineages). The expression levels of TNF, IL-6, and IL-10 were not markedly up-regulated in these four analyzed leukocyte lineages (Figure 2E–G). These results suggest that stress-induced pro-inflammatory changes associated with IL-1β involve neutrophils, macrophages, and monocytes.

**Figure 3:**
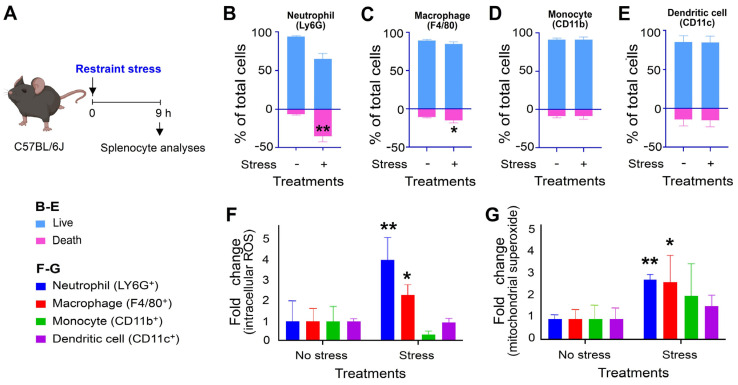
Relative levels of cell death, cellular ROS, and mitochondrial superoxide of splenic innate immune leukocytes. (**A**–**D**) Relative percentage of live and death cells of innate immune splenocytes, including (**A**) Ly6G^+^ neutrophil, (**B**) F4/80^+^ macrophage, (**C**) CD11b^+^ monocyte, (**D**) CD11c^+^ dendritic cell, in mice with (stress + groups) and without (stress − groups) stress. The levels of respective leukocyte lineages in the no stress groups were normalized to one-fold. (**E**–**G**) Relative levels of cellular ROS (**E**), and mitochondrial superoxide (**F**) without (no stress groups) and with (stress groups) stress in mice were analyzed. (**E**,**F**) The levels of respective leukocyte lineages in the no stress groups were normalized to one-fold. (**A**–**G**) * *p* > 0.05, ** *p* > 0.01, vs. respective no stress groups; *n* = 6 (3 experiments with a total of 6 mice per group). The mouse graph was created with BioRender.com, accessed on 1 January 2024.

The activation of leukocytes and subsequent inflammation can lead to cell death [33,34], which involves the production of ROS and increased mitochondrial burden [35,36]. To investigate whether stress-induced splenic changes involve cell death, ROS production, and mitochondrial burden, we analyzed the relative levels of these parameters in splenic neutrophils, macrophages, monocytes, and dendritic cells. Our findings revealed that only neutrophils exhibited significantly increased cell death levels after 9 h of stress (Figure 3A, neutrophil vs. Figure 3B, macrophages, Figure 3C, monocytes, and Figure 3D, dendritic cells). Consistent with the cell death measurements, ROS levels were up-regulated only in neutrophils among the four tested leukocyte lineages (Figure 3E). Despite the observation that both neutrophils and macrophages showed elevated levels of mitochondrial superoxide (Figure 3F), the outcomes indicate that neutrophils are the main leukocyte population in the spleen that undergo increased cellular ROS and cell death responses following restraint stress.

### 2.2. Increased under Stress, NET-Associated Cell Death Represents a Significant Regulated Cell Death Response in Splenic Neutrophils

**Figure 4:**
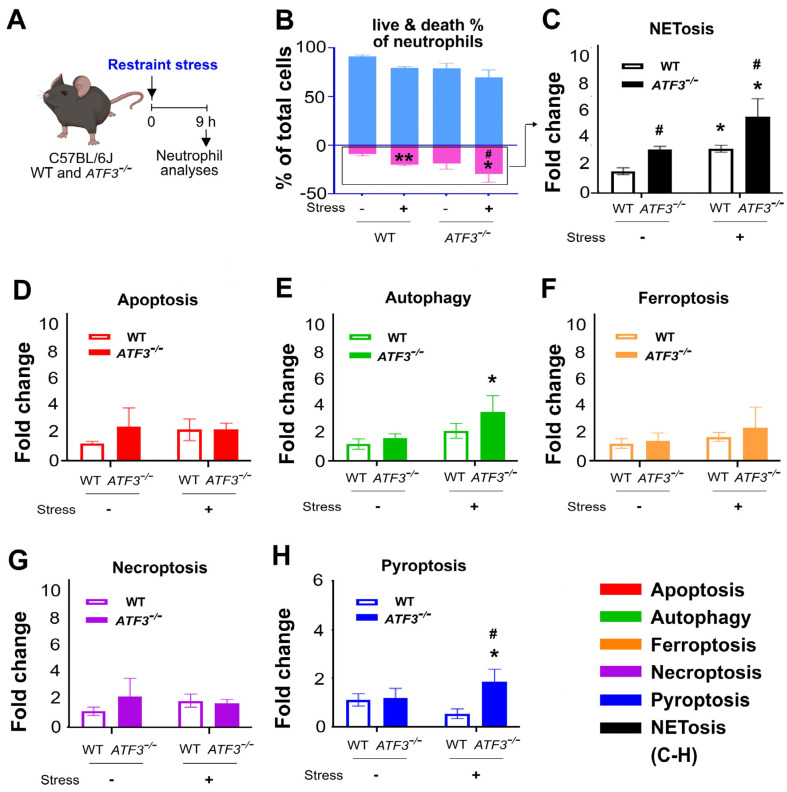
Restraint stress-induced cell death of splenic neutrophil in wild-type (*ATF3*^+/+^) and ATF3 deficient (*ATF3^−/−^*) mice. (**A**) Experiment outline. (**B**) Live and death cell percentage of splenic neutrophil from *ATF3*^+/+^ and *ATF3*^−/−^ mice without (stress − groups) and with (stress + groups) stress were analyzed by flow cytometry using Zombie NIR live/death analysis kit. Light blue represents the live cell population, while pink represents the dead cell population. (**C**–**H**) The regulated cell death (RCD) profiles, denoted by the alterations in the percentage of RCD-signal positive neutrophils in *ATF3*^+/+^ and *ATF3*^−/−^ mice under unstressed (stress − groups) and stressed (stress + groups) conditions, were assessed. The percentages of neutrophil RCD-marker^+^ cells, including NETosis (**C**), apoptosis (**D**), autophagy (**E**), ferroptosis (**F**), necroptosis (**G**), and pyroptosis (**H**), were delineated. In each group, the signal from wild-type mice was normalized to one-fold; *n* = 6 (3 experiments with a total of 6 mice per group); * *p* < 0.05, ** *p* < 0.01, significantly higher vs. without stress control groups; # *p* < 0.05, significantly higher vs. wild-type (WT) control groups. The mouse graph was created with BioRender.com, accessed on 1 January 2024.

There is evidence to suggest that a single cellular stressor may trigger multiple RCD responses [30,31,32,37]. In this study, we investigated whether restraint stress leads to multiple RCDs of splenic neutrophils, including NETosis, pyroptosis, autophagy, ferroptosis, necroptosis, and apoptosis. Our RCD profiling findings revealed that after stress, there was a marked increase in the levels of neutrophil NETosis in wild-type mice (Figure 4A, experiment outline; Figure 4B, % of live vs. death population; Figure 4C–H, NETosis, apoptosis, autophagy, ferroptosis, necroptosis, and pyroptosis; representative flow cytometry gatings in Figure S1).

### 2.3. ATF3 Deficiency Leads to an Exacerbated Stress-Induced NET Formation in Spleen Neutrophils

It has been shown that a deficiency of ATF3, an anti-inflammatory regulator, can worsen stress-induced GI leakage [15]. If ATF3 plays a role in reducing stress-induced GI injury associated with neutrophil NETosis, it is possible that the splenic neutrophils could activate ATF3. In addition, it is theoretically expected that the neutrophils from stressed *ATF3*^−/−^ mice may show increased levels of NET formation when compared to the wild-type controls. To test this hypothesis, the ATF3 expression and RCDs of splenic neutrophils from ATF3 deficient (*ATF3*^−/−^) and wild-type (*ATF3*^+/+^) mice were analyzed before and after stress. As suggested, here we observed increased expression levels of the ATF3 RNA of spleen Ly6G^+^ neutrophils (Figure S2). Additionally, restraint stress induced significantly higher levels of neutrophil cell death (Figure 4A experiment outline, Figure 4B % of live vs. death population) and NETosis marker detection (Figure 4C) in *ATF3*^−/−^ mice compared to wild-type mice. The levels of pyroptosis and autophagy were also higher in the neutrophils of *ATF3*^−/−^ mice than in those of wild-type mice. However, NET-associated cell death remained the major increased RCD response under stress (Figure 4E,H). We calculated the percentage of NETosis and other RCDs of splenic neutrophils using the methods described in Figure 4 to determine whether NETosis is the major RCD with the highest level of change. The analysis of outcomes revealed that, among the scrutinized forms of regulated cell death, NETosis emerged as the primary and significantly heightened response of neutrophils following restraint stress. This response was particularly exacerbated in ATF3-deficient mutant mice (Figure 4B,C).

**Figure 5:**
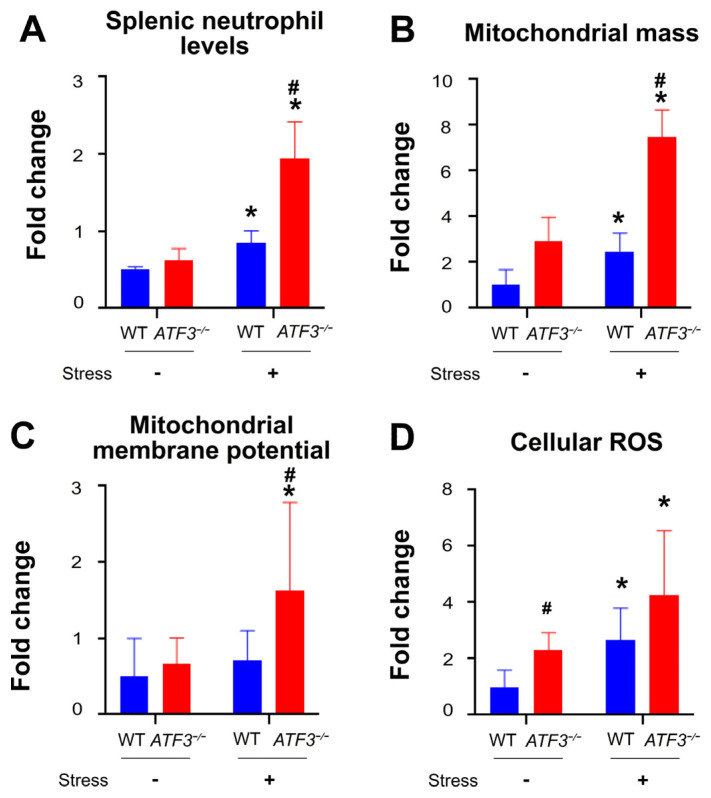
Restraint stress-induced changes in splenic neutrophil in wild-type (*ATF3*^+/+^) and ATF3 deficient (*ATF3^−/−^*) mice. (**A**) Relative cell abundance, (**B**) mitochondrial mass, (**C**) mitochondrial membrane potential, and (**D**) cellular ROS levels in the spleen neutrophils of *ATF3*^+/+^ and *ATF3*^−/−^ mice, with (stress + groups) and without (stress − groups) stress, were analyzed by flow cytometry. Blue denotes the no-stress groups, while red indicates the stressed groups. *n* = 6 (3 experiments with a total of 6 mice per group); * *p* < 0.05, vs. vehicle control (0 g/mL) groups; # *p* < 0.05, significantly higher vs. wild-type (WT) control groups.

Further investigation into the protective function of ATF3 involved assessing additional parameters, including relative cell abundance, mitochondrial mass, mitochondrial membrane potential, and cellular ROS levels in the spleen neutrophils of wild-type and ATF3 mutant mice. Our findings revealed that these pathological changes were all intensified in the ATF3 mutant mice compared to the wild-type mice (Figure 5A–D) (representative flow cytometry gatings in Figure S3; and fluorescent dyes, including 2′,7′-dichlorodihydrofluorescein diacetate (DCFDA), MitoTracker Green, MitoTracker Red CMXRos, and MitoSOX Red, were employed for flow cytometry analysis to assess cellular ROS, mitochondrial mass, mitochondrial membrane potential, and mitochondrial superoxide levels, respectively). These collective results suggest that the abnormal changes in relative cell abundance, mitochondrial mass, mitochondrial membrane potential, and the cellular ROS in spleen neutrophils are linked to the increased NETosis of spleen neutrophils after stress (Figure 5A–D).

### 2.4. Treatments of NETosis Inhibitor GSK484 Ameliorated Restraint Stress-Induced GI Leakage

**Figure 6:**
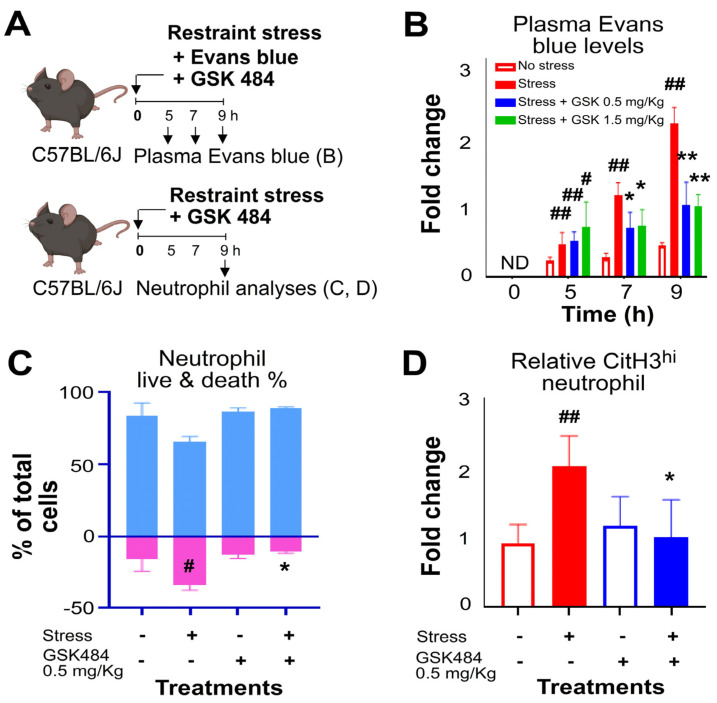
Treatments of NETosis inhibitor GSK484 rescued restraint stress-induced GI leakage and pro-inflammatory changes in splenic neutrophil in mice. (**A**) Experiment outline. (**B**) Plasma Evans blue levels, (**C**) neutrophil cell death levels, (**D**) neutrophil NETosis levels, in the spleen of mice, with (stress + groups) or without (stress − groups) stress, and with (GSK484 + groups) or without (GSK484 − groups) GSK484 treatments, were analyzed; *n* = 6 (3 experiments with a total of 6 mice per group); # *p* < 0.05, ## *p* < 0.01, exacerbated vs. no stress control groups; * *p* < 0.05, ** *p* < 0.01, rescued vs. vehicle control (0 g/mL, without GSK484 treatments) groups. (**B**) ND: not detected. (**C**) Light blue represents the live cell population, while pink represents the dead cell population. (**D**) The mouse graph was created with BioRender.com, accessed on 1 January 2024.

As NET formation may be involved in stress-induced GI injury, it could be a potential target for drug therapy to manage the disease. In our study, we found that treatment with the NET formation inhibitor GSK484 significantly suppressed stress-induced GI leakage (Figure 6A, experiment outline, Figure 6B), neutrophil cell death (Figure 6C), and neutrophil NET formation signal (Figure 6D). Intriguingly, although the commonly prescribed non-steroidal anti-inflammatory drug ibuprofen showed a tendency to alleviate restraint stress-induced GI leakage when compared, the difference was not statistically significant (Figure S4). In contrast, treatment with GSK484 at 0.5 mg/kg markedly ameliorated restraint stress-induced GI leakage in mice (Figure S4). These findings collectively suggest that stress-induced neutrophil NET formation is part of the pathological process of GI injury and can be improved by the use of the NET formation inhibitor GSK484.

## 3. Discussion

Studies have demonstrated that psychological stress can increase the number of leukocytes by mobilizing hematopoietic progenitor cells, which leads to persistent splenic myelopoiesis [38]. The restraint stress model is a common approach to measure behavioral and physiological responses induced by psychological stress, and it has been shown to result in abnormal immune regulation, including changes in splenic leukocyte counts, lymphocyte proliferation, and leukocyte redistribution [24]. Nevertheless, the exact cellular reactions of splenic leukocytes and their role in stress-induced GI injury remain incompletely elucidated. Psychological stress-induced gut injuries pose an intricate and ambiguously characterized challenge in present-day research.

Our findings indicate that both the total cell count and death-cell count of splenic innate leukocytes increase following restraint stress. Interestingly, this response is similar to the immune response observed in the mouse models of cecal ligation puncture (CLP) and other severe GI infections [39,40,41,42]. The CLP model involves the perforation of the cecum, which allows for the release of fecal matter into the peritoneal cavity, triggering an exaggerated immune response caused by polymicrobial infection [39]. This, in turn, leads to further cell death and leukocyte infiltration in the spleen [39,40,43]. While we did not detect any LPS in the mouse circulation after restraint stress, we did observe orally fed fluorescent-LPS labeled leukocytes present in the spleen (Figure S5). This suggests that, despite restraint stress-induced GI injuries not significantly elevating circulating LPS levels, bacterial components from the GI tract were still able to leak into the mouse body and become trapped in the spleen. Following restraint stress-induced GI leakage, these fecal materials may lead to subsequent cell death and leukocyte infiltration in the spleen, similar to the CLP model [39,40,43], albeit to a lesser extent.

Our findings in this report indicate that restraint stress primarily affects neutrophils, with NETosis being the main cell death response observed in splenic neutrophils. Neutrophil NET formation is a two-edged sword in innate immunity [44]. Neutrophils are the first line of defense against infections by releasing cytokines and anti-microbials, engulfing pathogens, and initiating NETosis, a cell death process that leads to NET formation [26,45]. However, excessive NET formation can also cause inflammatory pathophysiology, including GI injuries such as ischemia–reperfusion injury and inflammatory bowel disease (IBD) [44,46,47,48]. The role of NETs in acute psychological stress-induced GI injury is not clear. Our analysis revealed that restraint stress significantly increased cellular ROS and cell death levels in splenic neutrophils. NETosis is the primary cell death response of neutrophils after stress, among the analyzed RCD pathways. Treatment with the NETosis inhibitor GSK484 significantly reduced neutrophil cell death levels and GI leakage, indicating that neutrophil NETosis is involved in restraint stress-induced GI injury.

The RCD profiling experiments have provided evidence that a single type of cellular stress, such as a viral protein or nanodiamond particle, can trigger various forms of RCD in different cell types, including neutrophils [30,31,32,37]. The presence of elevated levels of multiple RCDs is termed a cell-type-specific RCD profile [30,31,32,37]. Given that restraint stress can lead to the induction of multiple neuroendocrine and pro-inflammatory cellular stressors, it is plausible that it can cause various RCDs in neutrophils. Inhibitors that target the major RCD pathways may have therapeutic potential for injuries caused by restraint stress. The rationale behind selecting GSK484, a peptidyl arginine deiminase 4 (PAD4) inhibitor, as the intervention of choice lies in its ability to target a key player in the NETosis pathway [49,50]. PAD4 is known to catalyze the conversion of arginine residues to citrulline, a crucial step in histone citrullination—a hallmark event in NETosis [51,52]. By inhibiting PAD4 activity, GSK484 effectively suppresses NETosis, thus attenuating the inflammatory response associated with excessive NET formation [32,49]. Our data analysis shows that NETosis GSK484 can alleviate stress-induced pro-inflammatory changes in splenic neutrophils and GI injury, indicating that stress-induced neutrophil NETosis and inflammation play a critical role in GI injury. Additionally, compared to wild-type (*ATF3*^+/+^) controls, neutrophils from *ATF3*^−/−^ mice displayed significantly higher levels of NETosis and IL-1 expression after restraint stress. These findings suggest that ATF3 is a natural regulator that helps rescue GI injury under stress.

ATF3 is a transcription factor that contains a basic region–leucine zipper DNA binding domain and functions as an anti-inflammatory regulator [17,53]. Previous reports have mainly focused on its anti-inflammatory effects in macrophages [17,54,55], and its roles in other types of leukocyte lineages are less well understood. Although evidence suggests that ATF3 regulates neutrophil migration and infiltration [56], its role in regulating neutrophil inflammation and NETosis remains unclear. In this study, we observed that stress-induced ATF3 expression in spleen neutrophils and ATF3 deficiency led to an increase in neutrophil NETosis cell death, which was associated with elevated levels of cellular ROS, changes in mitochondrial mass and membrane potential after stress. These results suggest that ATF3 has a profound anti-inflammatory role in neutrophils, and its protective effect against stress-induced GI injury involves the suppression of neutrophil activation and NETosis. In addition, previous studies have shown that chronic exposure to acute restraint stress can contribute to and worsen colitis and IBD-like pathological alterations [9,10,57,58]. This suggests that the pathogenesis of acute restraint stress could serve as an early stage of colitis and IBD-like disease. Given the limited understanding of effective treatments for these conditions, NETosis and ATF3 could also be viable targets for managing the initial progression of colitis and IBD-like disease.

In our previous study, we showed that administering the proton pump inhibitor esomeprazole alleviated acute gut leakage caused by restraint stress [15]. This implies that the mechanism of restraint stress-induced gut injury might involve increased gastric acid secretion and/or weakened resistance of the gut to gastric acid, resembling gastrointestinal ulcers in humans. Such stress-induced injuries likely further lead to subsequently inflammation and cell death. However, it is important to note that the effects of restraint stress may not entirely mirror those of typical human stressors. Furthermore, given the current uncertainty regarding the precise mechanism, further exploration is needed to understand how psychological stress contributes to the mechanism of gastrointestinal pathogenesis, the involvement of brain–gut cross talks, and how ATF3 regulation intersects with neutrophil inflammation, NET formation, alterations in inflammation factors such as IL-1, and gut injuries.

## 4. Materials and Methods

### 4.1. Laboratory Mice

Wild-type (WT) C57BL/6J mice aged 8–12 weeks were purchased from the National Laboratory Animal Center (Taipei, Taiwan) [59,60,61]. Genetically deficient *ATF3*^−/−^ (ATF3 gene knockout, KO) mice with a C57BL/6J background [15,62,63], were provided by Dr. Tsonwin Hai. *ATF3*^−/−^ mice were backcrossed with WT C57Bl/6J mice over six generations. The experimental mice were accommodated in the Animal Center of Tzu-Chi University, where they resided in a controlled environment that was pathogen-free, regulated for light and temperature, and maintained on a 12 h light to 12 h dark (12L:12D) cycle. The mice tested were all male, and no more than 5 mice lived in one cage. They had ad libitum access to both food and filtered water. Furthermore, the Animal Center administered veterinary care, conducted health monitoring, facilitated environmental enrichment, and implemented appropriate husbandry practices to safeguard the well-being and minimize the stress levels of the experimental animals. Approximately 360 wild-type mice and 80 *ATF3*^−/−^ mice were employed. All experimental protocols for examining the experimental animals were approved by the Animal Care and Use Committee of Tzu-Chi University, Hualien, Taiwan (approval ID: 110024).

### 4.2. Restraint Stress and Measurement of Stress-Induced GI Leakage

The Evans blue-fed restraint stress-induced GI leakage mouse model was performed according to previously described methods [15,16]. The restraint stress mouse model induces psychological stress in mice by confining them within specialized tubes, restricting their movement for a set duration [27]. This model, validated by changes in behavior post-stress, is extensively employed to investigate physiological and behavioral responses to stress and probe the mechanisms underlying stress-related disorders [27,64,65]. During restraint stress, mice undergo fluctuations in stress hormone levels [66,67]. To induce restraint stress [15,68,69], the mice were placed in a 50-mL plastic falcon tube for 9 h. To ensure sufficient air supply, holes were created at the tapering end of the falcon tube. After the stress challenge began, blood samples (50 µL) were collected during the experiment at 0, 5, 7 and 9 h. The mice were fed with Evans blue (1.2 g/kg, Santa Cruz Biotechnology, Santa Cruz, CA, USA), using a steel feeding tube just before the commencement of the stress challenge. Their blood sample was isolated by collecting blood in an Eppendorf tube and mixing it with an equal proportion of anti-coagulant citrate dextrose solution to prevent coagulation, and then plasma was obtained by centrifugation to pellet down the blood cells [30,31,32]. The collected plasma was transferred to 96 well plates, in which the concentration of Evans blue was determined using a full spectrum analyzer (Multiskan Spectrum, Thermo Fisher Scientific, Waltham, MA, USA) at 620 nm. The NETosis inhibitor (0.5 and 1.5 mg/kg GSK484; a protein arginine deiminase 4 inhibitor) was used to rescue stress-induced GI injury. GSK484 was prepared for intraperitoneal administration in mice by being dissolved in dimethyl sulfoxide (DMSO), with the final concentration of DMSO being 0.1% in 0.9% NaCl solution.

### 4.3. Flow Cytometry: Cell Death, NETosis, ROS, and Mitochondrial Analysis

To prevent the fluorescent quenching effect of Evans blue dye, flow cytometry analyses were performed using tissue and cell samples obtained from mice that were not exposed to Evans blue feeding. Mouse spleen samples were cut into tiny pieces and incubated with a collagenase D (Sigma-Aldrich, Burlington, MA, USA; 1 mg/mL)-containing serum-free cell culture medium for 30 min in a 15 mL falcon tube at 37 °C with shaking (upright shaking incubator; OSI 500, Kansin Instruments, New Taipei City, Taiwan) after 9 h of restraint stress, following the described methods [15]. Mouse splenocytes were dissociated from the remaining cell and tissue pellets by incubation with 2 mL of a non-enzymatic cell-dissociation solution (Sigma-Aldrich) for 10 min at room temperature. After washing (phosphate-buffered saline, PBS), the cells were subjected to fluorescence staining to determine the levels of RCDs, ROS and mitochondrial burden and fluorescent lipopolysaccharide (LPS). Various RCDs, including NETosis (fluorescent anti-citrullinated histone H3, CitH3 antibody, Abcam, Cambridge, UK), apoptosis (CaspGLOWTM Red Active Caspase-3 Staining Kit, BioVision, Milpitas, CA, USA), autophagy (Cyto-ID™ Autophagy Detection Kit, Enzo Life Sciences, Farmingdale, NY, USA), ferroptosis (C11 BODIPY 581/591, Cayman Chemical, Ann Arbor, MI, USA), necroptosis (RIP3/B-2 alexa Fluor 488, Santa Cruz Biotechnology, Santa Cruz, CA, USA), pyroptosis (Caspase-1 Assay, Green, ImmunoChemistry Technologies, Davis, CA, USA), and live/dead cell labeling (Zombie NIR Fixable Viability Kit, Biolegend, San Diego, CA, USA), were analyzed using respective cell labeling reagents (30 min in PBS). Notably, to avoid detecting those RCD signals not contributed by the purification and manipulation processes, Zombie-live/dead cell labeling (30 min) should be performed immediately after splenocyte isolation and before the subsequent RCD signal staining (30 min) then, the RCD pattern should be analyzed, focusing only on the dead-cell population indicated by Zombie-live/dead staining. To analyze the induction of cellular IL-1, ROS and mitochondrial superoxide levels, anti-IL-1 antibody (Biolegend, San Diego, CA, USA) and a fluorescent secondary antibody (Jackson Immunoresearch, West Grove, PA, USA), cell-permeant 2′,7′-dichlorodihydrofluorescein diacetate (DCFDA, cellular ROS; Thermo Fisher Scientific) MitoTracker Green (mitochondrial mass; Thermo Fisher Scientific), MitoTracker Red CMXRos (mitochondrial membrane potential; Thermo Fisher Scientific) and MitoSOX Red (mitochondrial superoxide indicator; Thermo Fisher Scientific), and fluorescent-LPS (oral-fed; Thermo Fisher Scientific) were used (30 min in PBS). For the staining of a non-cell permeable agent (e.g., anti-IL-1 antibody, fluorescent-LPS), splenocytes were fixed by 4% paraformaldehyde in PBS for 30 min, permeabilized by 0.1% Triton X-100 in 4% paraformaldehyde PBS for 10 min, and then blocked with 1% bovine serum albumin buffer for 30 min at room temperature, as previously described [70]. The samples were then analyzed using flow cytometry (Gallios, Beckman Coulter Life Sciences, Brea, CA, USA) for quantitative analysis [71,72,73].

### 4.4. Neutrophil Isolation and RNA-Level Analysis

For RNA isolation, cDNA preparation and reverse-transcription quantitative real-time polymerase chain reaction (RT-qPCR) analysis, spleen samples from the mice were isolated, washed (phosphate-buffered saline, PBS) and cut into tiny pieces and incubated with a collagenase D (Sigma-Aldrich, Burlington, MA, USA; 1 mg/mL)-containing PBS. Neutrophils were isolated using a mouse neutrophil isolation kit (Miltenyi Biotec, Bergisch Gladbach, North Rhine-Westphalia, Germany). After dissolving in Trizol (Ambion, Thermo Fisher Scientific) and the implementation of the ribonuclease-free deoxyribonuclease treatments and standard isolation protocols, the neutrophil RNA concentration was analyzed using a NanoDrop spectrophotometer (Thermo Fisher Scientific). The RNA (1 μg) was used to synthesize complementary DNA (cDNA) using an iScript cDNA Synthesis Kit (Bio-Rad Laboratories, Hercules, CA, USA). The obtained cDNA was stored at −20 °C before use. For qRT-PCR analysis, cDNA (2 µL) was mixed with 10 µL SYBR Green (Thermo Fisher Scientific), 0.5 µL of each of the forward and reverse primers, and 7 µL of ddH_2_O, and quantified in a real-time reverse-transcription linkage instrument (StepOnePlus Real-Time PCR System, Thermo Fisher Scientific) with varying annealing temperatures according to the primers. Each sample was run in triplicate and the average cycle threshold (Ct) values were used to calculate 2^−ΔΔCT^ with that of the internal control GAPDH (glyceraldehyde-3-phosphate dehydrogenase) gene expression following the described methods [15]. The primers used for the amplification of ATF3 are shown as follows: forward primer, 5′-AGT GAC AGC ATG AGC CCT CT-3′; and reverse primer, 5′-GCA GCA CTG ACC TGA TCA AA-3′.

### 4.5. Statistical Analyses

The experimental results were analyzed using Microsoft Office Excel 2003 and SPSS 17, and the results were reported as mean ± standard deviation (SD). The statistical significance of the obtained results was examined using a one-way analysis of variance and post hoc Bonferroni-corrected *t*-test; the probability of type 1 error α = 0.05 was considered the threshold of statistical significance.

## 5. Conclusions

In summary, we found that restraint stress impacts the innate immune leukocyte lineage of the spleen, specifically neutrophils. This leads to an increase in the expression of the pro-inflammatory cytokine IL-1, cellular ROS, and cell death. Neutrophil cell death, primarily through NETosis, occurs in the spleen. Additionally, NET formation contributes to the development of GI injury caused by psychological stress. By administering GSK484, an inhibitor of NETosis, we successfully mitigated stress-induced GI injury in mice. Moreover, ATF3 acts as a natural anti-inflammatory and prosurvival factor against NETosis. The absence of ATF3 worsens stress-induced neutrophil cell death and NETosis. These findings provide new insights into the mechanisms of stress-induced inflammation and could assist in developing innovative therapeutic approaches for treating stress-induced GI injury.

## Data Availability

The datasets used and analyzed during the current study are available from the corresponding author on reasonable request.

## Supplementary Materials

The following supporting information can be downloaded at: https://www.mdpi.com/article/10.3390/ijms25105261/s1.

## References

[1] Huerta-Franco M.R., Vargas-Luna M., Tienda P., Delgadillo-Holtfort I., Balleza-Ordaz M., Flores-Hernandez C. (2013). Effects of occupational stress on the gastrointestinal tract. World J. Gastrointest. Pathophysiol..

[2] Madison A., Kiecolt-Glaser J.K. (2019). Stress, depression, diet, and the gut microbiota: Human-bacteria interactions at the core of psychoneuroimmunology and nutrition. Curr. Opin. Behav. Sci..

[3] Sapolsky R.M. (2004). Why Zebras Don’t Get Ulcers.

[4] Oligschlaeger Y., Yadati T., Houben T., Condello Olivan C.M., Shiri-Sverdlov R. (2019). Inflammatory Bowel Disease: A Stressed “Gut/Feeling”. Cells.

[5] Rao M., Gershon M.D. (2016). The bowel and beyond: The enteric nervous system in neurological disorders. Nat. Rev. Gastroenterol. Hepatol..

[6] Disease G.B.D., Injury I., Prevalence C. (2018). Global, regional, and national incidence, prevalence, and years lived with disability for 354 diseases and injuries for 195 countries and territories, 1990–2017: A systematic analysis for the Global Burden of Disease Study 2017. Lancet.

[7] Long-Smith C., O’Riordan K.J., Clarke G., Stanton C., Dinan T.G., Cryan J.F. (2020). Microbiota-Gut-Brain Axis: New Therapeutic Opportunities. Annu. Rev. Pharmacol. Toxicol..

[8] Cryan J.F., O’Riordan K.J., Cowan C.S.M., Sandhu K.V., Bastiaanssen T.F.S., Boehme M., Codagnone M.G., Cussotto S., Fulling C., Golubeva A.V. (2019). The Microbiota-Gut-Brain Axis. Physiol. Rev..

[9] Gao X., Cao Q., Cheng Y., Zhao D., Wang Z., Yang H., Wu Q., You L., Wang Y., Lin Y. (2018). Chronic stress promotes colitis by disturbing the gut microbiota and triggering immune system response. Proc. Natl. Acad. Sci. USA.

[10] Mawdsley J.E., Rampton D.S. (2005). Psychological stress in IBD: New insights into pathogenic and therapeutic implications. Gut.

[11] La Torre D., Van Oudenhove L., Vanuytsel T., Verbeke K. (2023). Psychosocial stress-induced intestinal permeability in healthy humans: What is the evidence?. Neurobiol. Stress.

[12] Campos A.C., Fogaca M.V., Aguiar D.C., Guimaraes F.S. (2013). Animal models of anxiety disorders and stress. Braz. J. Psychiatry.

[13] Pare W.P., Glavin G.B. (1986). Restraint stress in biomedical research: A review. Neurosci. Biobehav. Rev..

[14] Glavin G.B., Pare W.P., Sandbak T., Bakke H.K., Murison R. (1994). Restraint stress in biomedical research: An update. Neurosci. Biobehav. Rev..

[15] Chuang D.J., Pethaperumal S., Siwakoti B., Chien H.J., Cheng C.F., Hung S.C., Lien T.S., Sun D.S., Chang H.H. (2021). Activating Transcription Factor 3 Protects against Restraint Stress-Induced Gastrointestinal Injury in Mice. Cells.

[16] Pethaperumal S., Hung S.C., Lien T.S., Sun D.S., Chang H.H. (2022). P-Selectin is a Critical Factor for Platelet-Mediated Protection on Restraint Stress-Induced Gastrointestinal Injury in Mice. Int. J. Mol. Sci..

[17] Ku H.C., Cheng C.F. (2020). Master Regulator Activating Transcription Factor 3 (ATF3) in Metabolic Homeostasis and Cancer. Front. Endocrinol..

[18] Margraf A., Zarbock A. (2019). Platelets in Inflammation and Resolution. J. Immunol..

[19] Huang H.S., Chang H.H. (2012). Platelets in inflammation and immune modulations: Functions beyond hemostasis. Arch. Immunol. Ther. Exp..

[20] Mebius R.E., Kraal G. (2005). Structure and function of the spleen. Nat. Rev. Immunol..

[21] Lewis S.M., Williams A., Eisenbarth S.C. (2019). Structure and function of the immune system in the spleen. Sci. Immunol..

[22] Bronte V., Pittet M.J. (2013). The spleen in local and systemic regulation of immunity. Immunity.

[23] Weinzirl J., Scheffers T., Garnitschnig L., Andrae L., Heusser P. (2020). Does the Spleen Have a Function in Digestion? Medical History, Phylogenetic and Embryological Development of the Splenogastric System. Complement. Med. Res..

[24] Jiang W., Li Y., Sun J., Li L., Li J.W., Zhang C., Huang C., Yang J., Kong G.Y., Li Z.F. (2017). Spleen contributes to restraint stress induced changes in blood leukocytes distribution. Sci. Rep..

[25] Yipp B.G., Kubes P. (2013). NETosis: How vital is it?. Blood.

[26] Papayannopoulos V. (2018). Neutrophil extracellular traps in immunity and disease. Nat. Rev. Immunol..

[27] Sun D.S., Lien T.S., Chang H.H. (2024). Restraint stress-associated gastrointestinal injury and implications from the Evans blue-fed restraint stress mouse model. Tzu Chi Med. J..

[28] Dhar S.K., Vishnupriyan K., Damodar S., Gujar S., Das M. (2021). IL-6 and IL-10 as predictors of disease severity in COVID-19 patients: Results from meta-analysis and regression. Heliyon.

[29] Timperi E., Barnaba V. (2020). Viral Hepatitides, Inflammation and Tumour Microenvironment. Adv. Exp. Med. Biol..

[30] Lien T.S., Sun D.S., Wu C.Y., Chang H.H. (2021). Exposure to Dengue Envelope Protein Domain III Induces Nlrp3 Inflammasome-Dependent Endothelial Dysfunction and Hemorrhage in Mice. Front. Immunol..

[31] Lien T.S., Chan H., Sun D.S., Wu J.C., Lin Y.Y., Lin G.L., Chang H.H. (2021). Exposure of Platelets to Dengue Virus and Envelope Protein Domain III Induces Nlrp3 Inflammasome-Dependent Platelet Cell Death and Thrombocytopenia in Mice. Front. Immunol..

[32] Lien T.S., Sun D.S., Hung S.C., Wu W.S., Chang H.H. (2021). Dengue Virus Envelope Protein Domain III Induces Nlrp3 Inflammasome-Dependent NETosis-Mediated Inflammation in Mice. Front. Immunol..

[33] Denning N.L., Aziz M., Gurien S.D., Wang P. (2019). DAMPs and NETs in Sepsis. Front. Immunol..

[34] Kolaczkowska E., Kubes P. (2013). Neutrophil recruitment and function in health and inflammation. Nat. Rev. Immunol..

[35] Schuiveling M., Vazirpanah N., Radstake T., Zimmermann M., Broen J.C.A. (2018). Metformin, A New Era for an Old Drug in the Treatment of Immune Mediated Disease?. Curr. Drug Targets.

[36] Forrester S.J., Kikuchi D.S., Hernandes M.S., Xu Q., Griendling K.K. (2018). Reactive Oxygen Species in Metabolic and Inflammatory Signaling. Circ. Res..

[37] Hung S.C., Ke L.C., Lien T.S., Huang H.S., Sun D.S., Cheng C.L., Chang H.H. (2022). Nanodiamond-Induced Thrombocytopenia in Mice Involve P-Selectin-Dependent Nlrp3 Inflammasome-Mediated Platelet Aggregation, Pyroptosis and Apoptosis. Front. Immunol..

[38] McKim D.B., Yin W., Wang Y., Cole S.W., Godbout J.P., Sheridan J.F. (2018). Social Stress Mobilizes Hematopoietic Stem Cells to Establish Persistent Splenic Myelopoiesis. Cell Rep..

[39] Toscano M.G., Ganea D., Gamero A.M. (2011). Cecal ligation puncture procedure. J. Vis. Exp..

[40] Turnbull I.R., Buchman T.G., Javadi P., Woolsey C.A., Hotchkiss R.S., Karl I.E., Coopersmith C.M. (2004). Age disproportionately increases sepsis-induced apoptosis in the spleen and gut epithelium. Shock.

[41] Li M., Liu B., Gu C., Zhang W., Yang J., Cheng G., Liu C., Hu X. (2017). Necroptosis of Splenic Macrophages Induced by Streptococcus gallolyticus subsp. Pasteurianus. Avian Dis..

[42] Ioannou M., Hoving D., Aramburu I.V., Temkin M.I., De Vasconcelos N.M., Tsourouktsoglou T.D., Wang Q., Boeing S., Goldstone R., Vernardis S. (2022). Microbe capture by splenic macrophages triggers sepsis via T cell-death-dependent neutrophil lifespan shortening. Nat. Commun..

[43] Sengupta S., Caldwell C.C., Nomellini V. (2020). Distinct Neutrophil Populations in the Spleen During PICS. Front. Immunol..

[44] Kaplan M.J., Radic M. (2012). Neutrophil extracellular traps: Double-edged swords of innate immunity. J. Immunol..

[45] Fuchs T.A., Abed U., Goosmann C., Hurwitz R., Schulze I., Wahn V., Weinrauch Y., Brinkmann V., Zychlinsky A. (2007). Novel cell death program leads to neutrophil extracellular traps. J. Cell Biol..

[46] Chen K., Shao L.H., Wang F., Shen X.F., Xia X.F., Kang X., Song P., Wang M., Lu X.F., Wang C. (2021). Netting Gut Disease: Neutrophil Extracellular Trap in Intestinal Pathology. Oxid. Med. Cell Longev..

[47] Drury B., Hardisty G., Gray R.D., Ho G.T. (2021). Neutrophil Extracellular Traps in Inflammatory Bowel Disease: Pathogenic Mechanisms and Clinical Translation. Cell Mol. Gastroenterol. Hepatol..

[48] Chen F., Liu Y., Shi Y., Zhang J., Liu X., Liu Z., Lv J., Leng Y. (2022). The emerging role of neutrophilic extracellular traps in intestinal disease. Gut Pathog..

[49] Lewis H.D., Liddle J., Coote J.E., Atkinson S.J., Barker M.D., Bax B.D., Bicker K.L., Bingham R.P., Campbell M., Chen Y.H. (2015). Inhibition of PAD4 activity is sufficient to disrupt mouse and human NET formation. Nat. Chem. Biol..

[50] Jaboury S., Wang K., O’Sullivan K.M., Ooi J.D., Ho G.Y. (2023). NETosis as an oncologic therapeutic target: A mini review. Front. Immunol..

[51] Rohrbach A.S., Slade D.J., Thompson P.R., Mowen K.A. (2012). Activation of PAD4 in NET formation. Front. Immunol..

[52] Liu X., Arfman T., Wichapong K., Reutelingsperger C.P.M., Voorberg J., Nicolaes G.A.F. (2021). PAD4 takes charge during neutrophil activation: Impact of PAD4 mediated NET formation on immune-mediated disease. J. Thromb. Haemost..

[53] Sun D.S., Chang H.H. (2021). Emerging role of the itaconate-mediated rescue of cellular metabolic stress. Tzu Chi Med. J..

[54] Lai P.F., Cheng C.F., Lin H., Tseng T.L., Chen H.H., Chen S.H. (2013). ATF3 Protects against LPS-Induced Inflammation in Mice via Inhibiting HMGB1 Expression. Evid.-Based Complement. Altern. Med..

[55] Hellmann J., Tang Y., Zhang M.J., Hai T., Bhatnagar A., Srivastava S., Spite M. (2015). Atf3 negatively regulates Ptgs2/Cox2 expression during acute inflammation. Prostaglandins Other Lipid Mediat..

[56] Boespflug N.D., Kumar S., McAlees J.W., Phelan J.D., Grimes H.L., Hoebe K., Hai T., Filippi M.D., Karp C.L. (2014). ATF3 is a novel regulator of mouse neutrophil migration. Blood.

[57] Israeli E., Hershcovici T., Berenshtein E., Zannineli G., Wengrower D., Weiss O., Chevion M., Goldin E. (2008). The effect of restraint stress on the normal colon and on intestinal inflammation in a model of experimental colitis. Dig. Dis. Sci..

[58] Koh S.J., Kim J.W., Kim B.G., Lee K.L., Kim J.S. (2015). Restraint stress induces and exacerbates intestinal inflammation in interleukin-10 deficient mice. World J. Gastroenterol..

[59] Ho Y.Y., Sun D.S., Chang H.H. (2020). Silver Nanoparticles Protect Skin from Ultraviolet B-Induced Damage in Mice. Int. J. Mol. Sci..

[60] Tsai C.L., Sun D.S., Su M.T., Lien T.S., Chen Y.H., Lin C.Y., Huang C.H., King C.C., Li C.R., Chen T.H. (2020). Suppressed humoral immunity is associated with dengue nonstructural protein NS1-elicited anti-death receptor antibody fractions in mice. Sci. Rep..

[61] Huang C.Y., Yu W.S., Liu G.C., Hung S.C., Chang J.H., Chang J.C., Cheng C.L., Sun D.S., Lin M.D., Lin W.Y. (2021). Opportunistic gill infection is associated with TiO_2_ nanoparticle-induced mortality in zebrafish. PLoS ONE.

[62] Cheng C.F., Ku H.C., Cheng J.J., Chao S.W., Li H.F., Lai P.F., Chang C.C., Don M.J., Chen H.H., Lin H. (2019). Adipocyte browning and resistance to obesity in mice is induced by expression of ATF3. Commun. Biol..

[63] Hartman M.G., Lu D., Kim M.L., Kociba G.J., Shukri T., Buteau J., Wang X., Frankel W.L., Guttridge D., Prentki M. (2004). Role for activating transcription factor 3 in stress-induced beta-cell apoptosis. Mol. Cell Biol..

[64] Xu Y.X., Liu G.Y., Ji Z.Z., Li Y.Y., Wang Y.L., Wu X.Y., Liu J.L., Ma D.X., Zhong M.K., Gao C.B. (2023). Restraint stress induced anxiety and sleep in mice. Front. Psychiatry.

[65] Li C.C., Munalisa R., Lee H.Y., Lien T.S., Chan H., Hung S.C., Sun D.S., Cheng C.F., Chang H.H. (2023). Restraint Stress-Induced Immunosuppression Is Associated with Concurrent Macrophage Pyroptosis Cell Death in Mice. Int. J. Mol. Sci..

[66] Gong S., Miao Y.L., Jiao G.Z., Sun M.J., Li H., Lin J., Luo M.J., Tan J.H. (2015). Dynamics and correlation of serum cortisol and corticosterone under different physiological or stressful conditions in mice. PLoS ONE.

[67] Nohara M., Tohei A., Sato T., Amao H. (2016). Evaluation of response to restraint stress by salivary corticosterone levels in adult male mice. J. Vet. Med. Sci..

[68] Zimprich A., Garrett L., Deussing J.M., Wotjak C.T., Fuchs H., Gailus-Durner V., de Angelis M.H., Wurst W., Holter S.M. (2014). A robust and reliable non-invasive test for stress responsivity in mice. Front. Behav. Neurosci..

[69] Chu X., Zhou Y., Hu Z., Lou J., Song W., Li J., Liang X., Chen C., Wang S., Yang B. (2016). 24-hour-restraint stress induces long-term depressive-like phenotypes in mice. Sci. Rep..

[70] Chang Y.S., Ko B.H., Ju J.C., Chang H.H., Huang S.H., Lin C.W. (2020). SARS Unique Domain (SUD) of Severe Acute Respiratory Syndrome Coronavirus Induces NLRP3 Inflammasome-Dependent CXCL10-Mediated Pulmonary Inflammation. Int. J. Mol. Sci..

[71] Wang T.F., Lin G.L., Chu S.C., Chen C.C., Liou Y.S., Chang H.H., Sun D.S. (2021). AQP0 is a novel surface marker for deciphering abnormal erythropoiesis. Stem Cell Res. Ther..

[72] Mandal J.P., Shiue C.N., Chen Y.C., Lee M.C., Yang H.H., Chang H.H., Hu C.T., Liao P.C., Hui L.C., You R.I. (2021). PKCdelta mediates mitochondrial ROS generation and oxidation of HSP60 to relieve RKIP inhibition on MAPK pathway for HCC progression. Free Radic. Biol. Med..

[73] Chen T.L., Chiang Y.W., Lin G.L., Chang H.H., Lien T.S., Sheh M.H., Sun D.S. (2018). Different effects of granulocyte colony-stimulating factor and erythropoietin on erythropoiesis. Stem Cell Res. Ther..

