# Supplementary material for: Restraint Stress-Induced Neutrophil Inflammation Contributes to Concurrent Gastrointestinal Injury in Mice
Source: Int J Mol Sci. 2024 May 11;25(10):5261. doi: 10.3390/ijms25105261 (PMC11121713; doi:10.3390/ijms25105261)
Supplement: Supplementary file 1 [file ijms-25-05261-s001.zip › ijms-2990937-supplementary.pdf]

Supplemental information for:

Restraint stress-induced neutrophil inflammation contributes to concurrent gastrointestinal injury in mice

Rina Munalisa[1], Te-Sheng Lien[1], Der-Shan Sun [1], Ching-Feng Cheng [2,3], Wen-Sheng Wu [4,5], Chi-Cheng Li [6,7], Chi-Tan Hu [8], Kuo-Wang Tsai [9], Yungling Leo Lee [10,11], Yu-Chi Chou [12], and Hsin-Hou Chang[1] *

[1] Department of Molecular Biology and Human Genetics, Tzu-Chi University, Hualien 970, Taiwan.

[2] Department of Pediatrics, Taipei Tzu Chi Hospital, Buddhist Tzu Chi Medical Foundation, Taipei, Taiwan

[3] Institute of Biomedical Sciences, Academia Sinica, Taipei, Taiwan

[4] Division of General Surgery, Department of Surgery, Hualien Tzu Chi Hospital, Buddhist Tzu Chi Medical Foundation Hualien 97004, Taiwan.

[5] Department of Laboratory Medicine and Biotechnology, College of Medicine, Tzu Chi University Hualien 97004, Taiwan.

[6] Department of Hematology and Oncology, Buddhist Tzu Chi General Hospital, Hualien, Taiwan

[7] Center of Stem Cell & Precision Medicine, Hualien Tzu Chi Hospital, Hualien, Taiwan

[8] Research Center for Hepatology, and Department of Gastroenterology, Buddhist Tzu Chi General Hospital, Buddhist Tzu Chi Medical Foundation, Hualien, Taiwan

[9] Department of Research, Taipei Tzu Chi Hospital, Buddhist Tzu Chi Medical Foundation, New Taipei City, Taiwan

[10] Institute of Biomedical Sciences, Academia Sinica, Taipei, Taiwan

[11] College of Public Health, China Medical University, Taichung, Taiwan

[12] Biomedical Translation Research Center, Academia Sinica, Taiwan

Running title: NETs contribute to stress-induced GI injury

* To whom correspondence and reprint requests should be addressed.
Hsin-Hou Chang Ph.D.
Room D407, Tzu-Chi University, No. 701, Section 3, Chung-Yang Road, Hualien 97004, Taiwan.
Tel: 886-3-8565301 ext 2667. Fax: 886-3-8578386.
E-mail: hhchang@mail.tcu.edu.tw

Pages 1-5: Figures S1, to S5

Figure S1

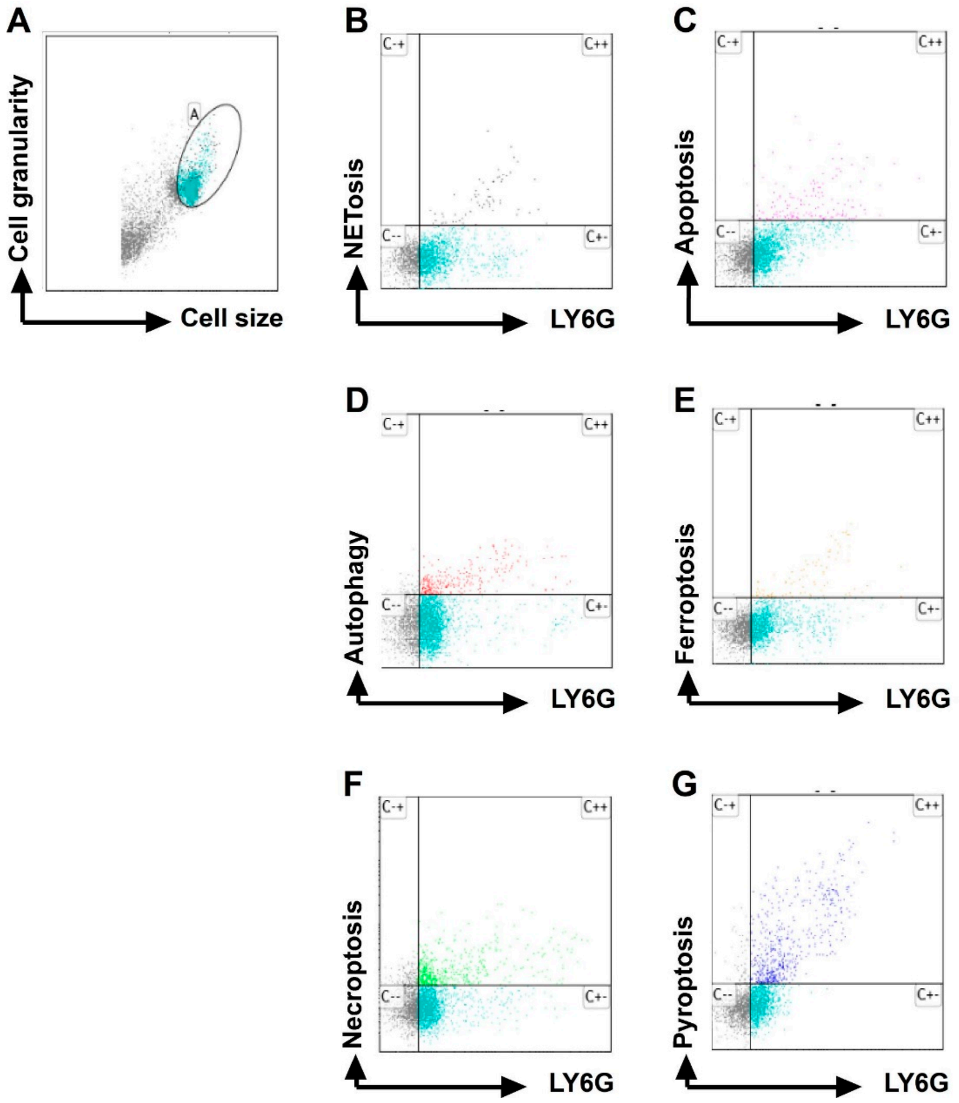

Figure S1. Representative flow cytometry plots demonstrating gating strategies for mouse splenic neutrophils. (A) Total splenic leukocytes gated based on cell granularity and size. (B to G) Neutrophils were then identified by co-staining with the neutrophil marker LY6G (LY6G^{+}) and specific dyes (the respective labaling kits are listed in the methods section) for NETosis (B), apoptosis (C), autophagy (D), ferroptosis (E), necroptosis (F), and pyroptosis (G). This allows differentiation of changes in these parameters in splenic neutrophils with or without restraint stress challenges.

Figure S2.

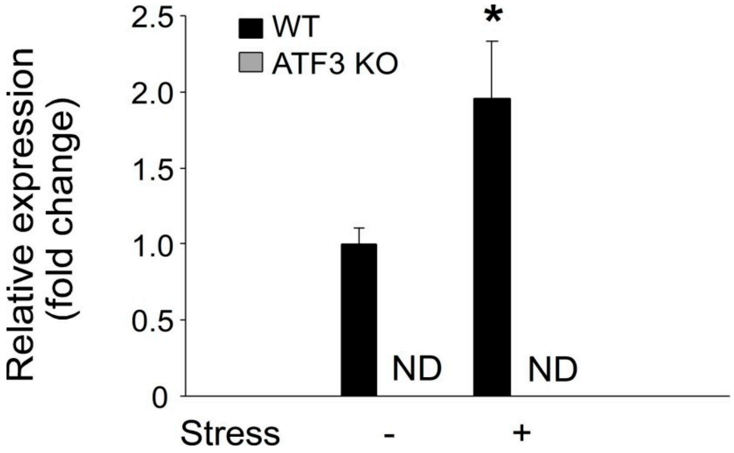

Figure S2. Relative spleen neutrophil mRNA expression levels of *ATF3* gene in wild type (*ATF3*$^{+/+}$) and ATF3 gene knockout (KO; *ATF3*$^{-/-}$) mice are indicated. Through qRT-PCR analysis, the relative mRNA expression levels of ATF gene of mouse neutrophil with or without 9 h restraint stress. The mRNA expression levels of control (no stress; stress -) groups were normalized to 1 fold. n = 3 (2 experiments with total 3 mice per group). * $P < 0.05$, vs. no stress control groups. ND: no detectable signal.

Figure S3

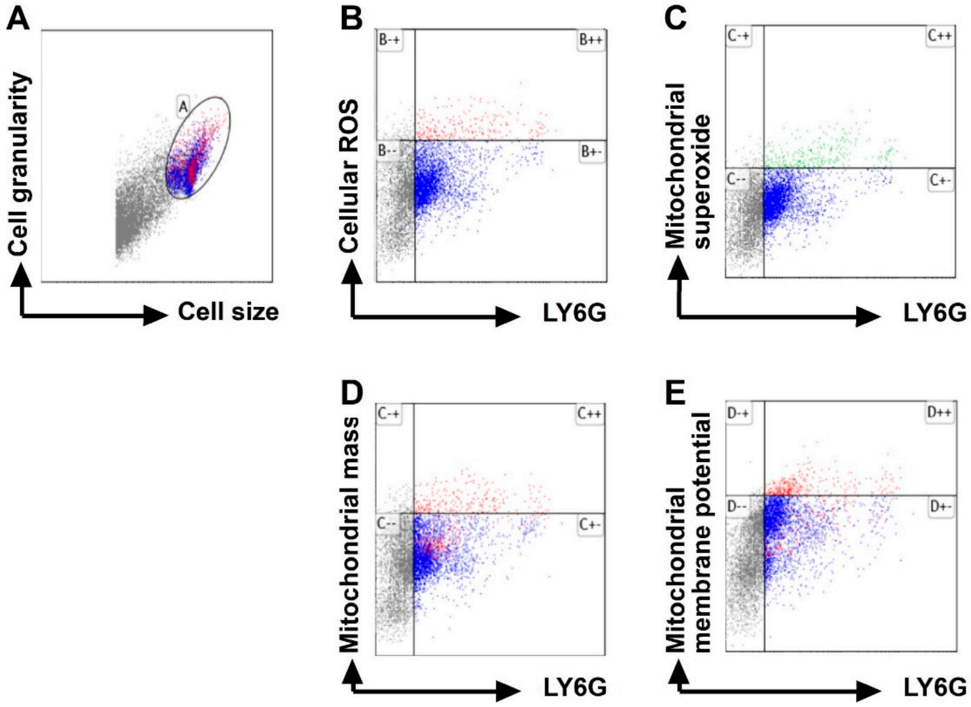

Figure S3. Representative flow cytometry plots demonstrating gating strategies for mouse splenic neutrophils. (A) Total splenic leukocytes gated based on cell granularity and size. (B to E) Neutrophils were then identified by co-staining with the neutrophil marker LY6G (LY6G$^+$) and specific dyes for cellular reactive oxygen species (ROS) (B), mitochondrial superoxide (C), mitochondrial mass (D), and mitochondrial membrane potential (E). This allows differentiation of changes in these parameters in splenic neutrophils with or without restraint stress challenges.

Figure S4.

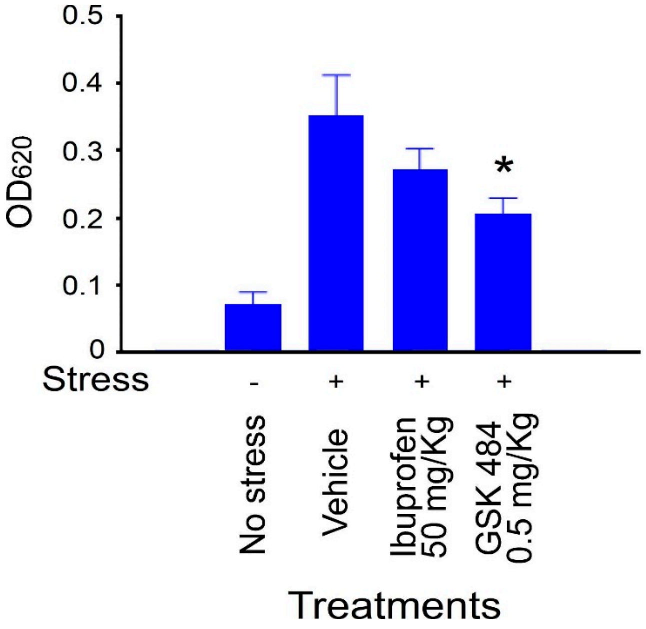

Figure S4. NETosis inhibitor GSK484 effectively rescues restraint stress-induced GI leakage versus ibuprofen control. Relative plasma levels of Evans blue were measured to assess the extent of stress-induced GI leakage and injury in mice. Both ibuprofen and GSK484 were administered to mice intraperitoneally. The dosage of ibuprofen at 50 mg/kg was selected based on previous reports (DOI: 10.1016/j.phrs.2013.04.009; DOI: 10.1111/j.1348-0421.2010.00249.x; DOI: 10.52794/hujpharm.962540). Control groups without stress were labeled as stress - while stressed groups were labeled as stress +. Each group consisted of 3 mice (2 experiments with a total of 3 mice per group). * Indicates statistical significance at $P < 0.05$ compared to stressed vehicle control groups.

Figure S5

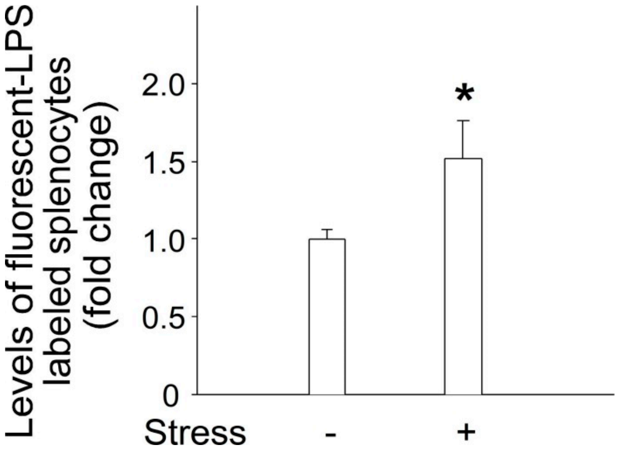

Figure S5. Levels of fluorescent-lipopolysaccharide (LPS) labeled splenocytes. Using flow cytometry, we aimed to determine if orally-fed fluorescent LPS could be captured by splenocytes. We normalized the average percentage of fluorescent-LPS labeled splenocytes in the control group (no stress; stress -) to 1-fold. n = 3 (2 experiments with total 3 mice per group). * $P < 0.05$, vs. no stress control groups. ND: no detectable signal.
